# Supplementary material for: Sweetness of Chilean Infants’ Diets: Methodology and Description
Source: Nutrients. 2022 Mar 30;14(7):1447. doi: 10.3390/nu14071447 (PMC9003557; doi:10.3390/nu14071447)
Supplement: Supplementary file 1 [file nutrients-14-01447-s001.zip › Edited Table S4.pdf]

**Table S4.** Database of food sweetness intensity, by food group

| No. | Product | Food Group | Flavor | Brand | Sweetness intensity value | Source | Number of products represented* |
|---|---|---|---|---|---|---|---|
| 1 | Chopped meals, savory commercial | Baby foods | Different flavors | Nestle | 3.7 | Sensory panel | 7 |
| 2 | Formula NAN, different subtypes | Baby foods | None | Nestle | 8.5 | Sensory panel | 6 |
| 3 | Formula Nutrilon, different subtypes | Baby foods | None | Nutricia | 8.5 | Sensory panel | 3 |
| 4 | Formula Progress gold 3 | Baby foods | Vanilla | Wyeth | 8.5 | Sensory panel | 1 |
| 5 | Formula S-26 Comfort Gold | Baby foods | None | Wyeth | 8.5 | Sensory panel | 1 |
| 6 | Formula Similac, different subtypes | Baby foods | None | Abbott | 8.5 | Sensory panel | 4 |
| 7 | Formula, different subtypes | Baby foods | None | Mead Johnson | 8.5 | Sensory panel | 4 |
| 8 | Instant Infant Cereal | Baby foods | Strawberry, wheat and other flavors | Nestum / Nestle | 18.7 | Sensory panel | 13 |
| 9 | Milk grown with sugar | Baby foods | Different flavors | Different brands | 32 | van Langeveld | 3 |
| 10 | Milk NIDAL, different subtypes | Baby foods | None | Nestle | 8.5 | Sensory panel | 2 |
| 11 | Milk, human | Baby foods | None | None | 13.5 | Mc Daniel | 1 |
| 12 | Milk, Purita Cereal powder | Baby foods | None | MINSAL® | 8.5 | Sensory panel | 1 |
| 13 | Milk, Purita Fortified milk powder | Baby foods | None | MINSAL® | 8.5 | Sensory panel | 1 |
| 14 | My soup | Baby foods | Bovine | MINSAL® | 2.9 | Sensory panel | 1 |
| 15 | Omega-3 Oil | Baby foods | None | Always happy | 1 | van Langeveld | 2 |
| 16 | Purees, fruit | Baby foods | Different flavors | Parent's Choice | 37.5 | Sensory panel | 5 |
| 17 | Purees, NaturNes | Baby foods | Organic peach | Nestle | 37.5 | Sensory panel | 1 |
| 18 | Purees, savory commercial | Baby foods | Different flavors | Nestle | 3.7 | Sensory panel | 6 |
| 19 | Sugar | Sugars and candy | None | Different brands | 58 | van Langeveld | 11 |
| 20 | Sugar, brown | Sugars and candy | None | Without brand | 58 | van Langeveld | 1 |
| 21 | Candy, Sunny | Sugars and candy | None | Calaf | 66 | van Langeveld | 1 |
| 22 | Candy | Sugars and candy | None | Without brand | 46 | van Langeveld | 1 |
| 23 | Candy, Sweet Tree | Sugars and candy | None | Ambrosoli | 46 | van Langeveld | 1 |
| 24 | Chewing gum, Big Time and Two-in-One | Sugars and candy | Mint | Different brands | 29 | van Langeveld | 1 |
| 25 | Chewing gum, Poosh | Sugars and candy | Strawberry | Arcor | 58 | van Langeveld | 1 |

| | | | | | | | |
|---|---|---|---|---|---|---|---|
| 26 | Chocolate, Toblerone, Costanuss, and Sahne nuss | Sugars and candy | Chocolate with nuts | Different brands | 65 | van Langeveld | 3 |
| 27 | Chocolate, 70% cocoa | Sugars and candy | Chocolate | Cacao soul | 44 | van Langeveld | 1 |
| 28 | Chocolate, bonbon | Sugars and candy | None | Without brand | 44 | van Langeveld | 1 |
| 29 | Chocolate, Chubi | Sugars and candy | None | Arcor | 60 | van Langeveld | 1 |
| 30 | Chocolate, filled | Sugars and candy | Different flavors | Different brands | 64 | van Langeveld | 6 |
| 31 | Chocolate, Loly choc | Sugars and candy | None | Ambrosoli | 55 | van Langeveld | 1 |
| 32 | Chocolate, Crispy Rolls | Sugars and candy | None | Costa | 59 | van Langeveld | 1 |
| 33 | Chocolate, Sapito, Rocklets | Sugars and candy | None | Arcor | 59 | van Langeveld | 2 |
| 34 | Chocolate, Trencito and Trencito balls | Sugars and candy | None | Nestle | 55 | van Langeveld | 2 |
| 35 | Lollipops | Sugars and candy | None | Different brands | 46 | van Langeveld | 14 |
| 36 | Chocolate cover | Sugars and candy | None | Without brand | 66 | van Langeveld | 1 |
| 37 | Quince sweet | Sugars and candy | Quince | Different brands | 74 | van Langeveld | 2 |
| 38 | Chewy sweets | Sugars and candy | Different flavors | Different brands | 58 | van Langeveld | 5 |
| 39 | Gummies | Sugars and candy | Different flavors | Different brands | 49 | van Langeveld | 9 |
| 40 | Ice cream, water- or fruit-based | Sugars and candy | Different flavors | Different brands | 33.8 | Martin | 5 |
| 41 | Ice cream | Sugars and candy | Different flavors | Different brands | 46 | van Langeveld | 3 |
| 42 | Chocolate, eggs | Sugars and candy | None | Different brands | 55 | van Langeveld | 2 |
| 43 | Jelly | Sugars and candy | Different flavors | Different brands | 49.2 | Sensory panel | 37 |
| 44 | Manjar / Dulce de leche (caramel) | Sugars and candy | None | Without brand | 81.1 | Sensory panel | 7 |
| 45 | Marshmallows | Sugars and candy | None | Different brands | 65 | van Langeveld | 3 |
| 46 | Jam regular or light | Sugars and candy | Different flavors | Different brands | 74 | van Langeveld | 12 |
| 47 | Honey | Sugars and candy | None | Without brand | 76 | van Langeveld | 1 |
| 48 | Mini jellies | Sugars and candy | Assorted flavors | Mabú | 49.2 | Sensory panel | 1 |
| 49 | Wafer, Bachata and Kilatte | Sugars and candy | Coconut, Choco cream, chocolate | Fruna | 59 | van Langeveld | 4 |

| | | | | | | | |
|---|---|---|---|---|---|---|---|
| 50 | Wafer, Bon o bon and Three Little Blacks Two in One | Sugars and candy | Different flavors | Arcor | 59 | van Langeveld | 3 |
| 51 | Wafer, Kit Kat, Super 8 | Sugars and candy | Chocolate | Nestle | 59 | van Langeveld | 2 |
| 52 | Wafer, Yours | Sugars and candy | None | Costa | 59 | van Langeveld | 2 |
| 53 | Dessert, Flan caramel sauce | Sugars and candy | Vanilla | Caricia | 33 | van Langeveld | 1 |
| 54 | Flavoring | Sugars and candy | Different flavors | Different brands | 65 | van Langeveld | 25 |
| 55 | Saccharin | Sugars and candy | None | Daily | 36.1 | Sensory panel | 1 |
| 56 | Sweet chocolate sauce | Sugars and candy | Chocolate | Gourmet | 74 | van Langeveld | 1 |
| 57 | Stevia | Sugars and candy | None | Different brands | 36.1 | Sensory panel | 11 |
| 58 | Sucralose | Sugars and candy | None | Daily | 36.1 | Sensory panel | 1 |
| 59 | Coffee, Ecco and Nescafé Tradition | Beverages | None | Nestle | 2 | van Langeveld | 2 |
| 60 | Flavored water, Aquarius | Beverages | Different flavors | The Coca Cola Company | 35 | van Langeveld | 6 |
| 61 | Flavored waters without sugar | Beverages | Apple | Lider | 35 | van Langeveld | 1 |
| 62 | Flavored waters without sugar | Beverages | Different flavors | Vivo | 35 | van Langeveld | 2 |
| 63 | Flavored waters without sugar, Benedictino | Beverages | Strawberry | The Coca Cola Company | 40 | van Langeveld | 1 |
| 64 | Flavored waters without sugar, Cachantun mas | Beverages | Different flavors | CCU | 35 | van Langeveld | 3 |
| 65 | Flavored waters without sugar, Daily | Beverages | Apple | Daily | 35 | van Langeveld | 1 |
| 66 | Flavored waters without sugar, Next | Beverages | Lemon | Soprole | 35 | van Langeveld | 1 |
| 67 | Herbs infusion, Chamomile, Natural Herbs, and Good herb | Beverages | None | Different brands | 4 | van Langeveld | 6 |
| 68 | Herbs infusion, Eight Herbs, and Rosehip | Beverages | Different flavors | Supremo | 4 | van Langeveld | 2 |
| 69 | Herbs infusion, Mint | Beverages | None | Different brands | 4 | van Langeveld | 2 |
| 70 | Instant powder drink with sugar | Beverages | Banana orange | Different brands | 31 | van Langeveld | 2 |
| 71 | Instant Powder Drink with sugar | Beverages | Pineapple | Different brands | 53 | van Langeveld | 14 |
| 72 | Instant Powder Drink with sugar | Beverages | Apple | Without brand | 53 | van Langeveld | 1 |
| 73 | Instant Powder Drink with sugar | Beverages | Peach | Different brands | 53 | van Langeveld | 2 |
| 74 | Instant Powder Drink with sugar | Beverages | Orange | Different brands | 31 | van Langeveld | 2 |

| | | | | | | | |
|---|---|---|---|---|---|---|---|
| 75 | Instant Powder Drink with sugar | Beverages | Strawberry | Different brands | 40 | van Langeveld | 3 |
| 76 | Instant Powder Drink without sugar | Beverages | Lemonade | Different brands | 53 | van Langeveld | 13 |
| 77 | Instant Powder Drink without sugar | Beverages | Strawberry, Berries | Different brands | 40 | van Langeveld | 14 |
| 78 | Instant Powder Drink without sugar | Beverages | Peach | Zuko | 53 | van Langeveld | 1 |
| 79 | Instant Powder Drink without sugar | Beverages | Pineapple | Different brands | 53 | van Langeveld | 3 |
| 80 | Instant Powder Drink without sugar | Beverages | Orange | Different brands | 31 | van Langeveld | 4 |
| 81 | Instant Powder Drink without sugar | Beverages | Tuna melon | Zuko | 53 | van Langeveld | 1 |
| 82 | Instant Powder Drink without sugar | Beverages | Orange | Without brand | 31 | van Langeveld | 1 |
| 83 | Mineral water without gas | Beverages | None | Without brand | 2 | van Langeveld | 1 |
| 84 | Mineral water without gas | Beverages | None | Puyehue | 2 | van Langeveld | 1 |
| 85 | Mineral water without gas | Beverages | None | Manantial | 2 | van Langeveld | 1 |
| 86 | Mineral water without gas, Benedictino and Vital | Beverages | None | The Coca Cola Company | 2 | van Langeveld | 2 |
| 87 | Mineral water without gas, Cachantun | Beverages | None | CCU | 2 | van Langeveld | 1 |
| 88 | Mineral water without gas, Pure life | Beverages | None | Nestle | 2 | van Langeveld | 1 |
| 89 | Natural juice with sugar, home made | Beverages | Different fruits, except apple | Without brand | 40 | van Langeveld | 6 |
| 90 | Natural juice with sugar, home made | Beverages | Apple, Green apple | Without brand | 35 | van Langeveld | 2 |
| 91 | Natural juice without sugar, home made | Beverages | Different fruits, except orange, lemon and apple | Without brand | 40 | van Langeveld | 15 |
| 92 | Natural juice without sugar, home made | Beverages | Orange, Lemon | Without brand | 31 | van Langeveld | 3 |
| 93 | Natural juice without sugar, home made | Beverages | Apple | Without brand | 35 | van Langeveld | 1 |
| 94 | Natural juice without sugar, home made | Beverages | Beet | Without brand | 9 | van Langeveld | 1 |
| 95 | Natural juice, commercial without sugar | Beverages | Pear, Mango | Guallarauco | 40 | van Langeveld | 2 |
| 96 | Natural juice, commercial without sugar | Beverages | Pear | Afe | 40 | van Langeveld | 1 |
| 97 | Nectar with sugar | Beverages | Peach | Different brands | 40 | van Langeveld | 8 |
| 98 | Nectar with sugar | Beverages | Apple | Different brands | 35 | van Langeveld | 6 |

| | | | | | | | |
|---|---|---|---|---|---|---|---|
| 99 | Nectar with sugar | Beverages | Orange | Different brands | 31 | van Langeveld | 5 |
| 100 | Nectar with sugar | Beverages | Apricot | Different brands | 40 | van Langeveld | 9 |
| 101 | Nectar with sugar | Beverages | Raspberry, Strawberry, Blueberry | Different brands | 40 | van Langeveld | 4 |
| 102 | Nectar with sugar | Beverages | Pineapple | Different brands | 40 | van Langeveld | 5 |
| 103 | Nectar with sugar | Beverages | Pear | Different brands | 40 | van Langeveld | 3 |
| 104 | Nectar without sugar | Beverages | Peach | Different brands | 40 | van Langeveld | 8 |
| 105 | Nectar without sugar | Beverages | Apricot | Without brand | 40 | van Langeveld | 1 |
| 106 | Nectar without sugar | Beverages | Apple | Different brands | 40 | van Langeveld | 3 |
| 107 | Nectar without sugar | Beverages | Orange | Different brands | 31 | van Langeveld | 5 |
| 108 | Nectar without sugar | Beverages | Frutifrutilla | Livean | 40 | van Langeveld | 1 |
| 109 | Ron | Beverages | None | Without brand | 7 | van Langeveld | 1 |
| 110 | Soft drink, 7 Up | Beverages | None | CCU | 51 | van Langeveld | 1 |
| 111 | Soft drink, Bilz, Bilz light, and Bilz zero | Beverages | None | Bilz & Pap | 51 | van Langeveld | 3 |
| 112 | Soft drink, Coca Cola | Beverages | None | The Coca Cola Company | 41 | van Langeveld | 1 |
| 113 | Soft drink, Coke life, Coke diet, and Coke zero | Beverages | None | The Coca Cola Company | 46 | van Langeveld | 3 |
| 114 | Soft drink, Fanta and Fanta zero | Beverages | Orange | The Coca Cola Company | 31 | van Langeveld | 2 |
| 115 | Soft drink, Frucola | Beverages | Cola | Fruna | 41 | van Langeveld | 1 |
| 116 | Soft drink, Inka cola | Beverages | Cola | The Coca Cola Company | 51 | van Langeveld | 1 |
| 117 | Soft drink, Kem and Kem zero | Beverages | Pineapple | CCU | 51 | van Langeveld | 2 |
| 118 | Soft drink, Lemon | Beverages | None | CCU | 51 | van Langeveld | 1 |
| 119 | Soft drink, Orange | Beverages | Orange | Fruna | 31 | van Langeveld | 1 |
| 120 | Soft drink, Orange crush | Beverages | Orange | CCU | 31 | van Langeveld | 1 |
| 121 | Soft drink, Pap and Pap zero | Beverages | None | Bilz & Pap | 51 | van Langeveld | 2 |
| 122 | Soft drink, Papaya | Beverages | Papaya | Without brand | 51 | van Langeveld | 1 |
| 123 | Soft drink, Pepsi | Beverages | None | CCU | 41 | van Langeveld | 1 |
| 124 | Soft drink, Pepsi zero | Beverages | None | CCU | 46 | van Langeveld | 1 |

| | | | | | | | |
|---|---|---|---|---|---|---|---|
| 125 | Soft drink, Pineapple | Beverages | Pineapple | Fruna | 51 | van Langeveld | 1 |
| 126 | Soft drink, Sprite | Beverages | None | The Coca Cola Company | 51 | van Langeveld | 1 |
| 127 | Sparkling mineral water | Beverages | None | Without brand | 2 | van Langeveld | 1 |
| 128 | Sparkling mineral water, Benedictino and Vital | Beverages | None | The Coca Cola Company | 2 | van Langeveld | 2 |
| 129 | Sparkling mineral water, Cachantun | Beverages | None | CCU | 2 | van Langeveld | 1 |
| 130 | Sportsmen drink, Gatorade | Beverages | Cool blue | CCU | 56 | van Langeveld | 1 |
| 131 | Tap water | Beverages | None | Without brand | 1 | van Langeveld | 1 |
| 132 | Tea, packaged liquid | Beverages | Grape | Without brand | 39 | van Langeveld | 1 |
| 133 | Tea/Black tea | Beverages | None | Different brands | 4 | van Langeveld | 15 |
| 134 | White wine | Beverages | None | Emblem Tea | 12 | van Langeveld | 1 |
| 135 | Cheese sticks | Fast food chains | None | Papa John's | 10.3 | Martin | 1 |
| 136 | Cheeseburger | Fast food chains | None | MC Donald's | 10.3 | Martin | 1 |
| 137 | Chicken, Fried breaded | Fast food chains | None | Kentucky fried chicken | 7 | van Langeveld | 1 |
| 138 | Chicken nugget | Fast food chains | None | MC Donald's | 7 | van Langeveld | 1 |
| 139 | Chicken sandwich | Fast food chains | None | MC Donald's | 4 | van Langeveld | 1 |
| 140 | Duchess potatoes | Fast food chains | None | MC Donald's | 7 | van Langeveld | 1 |
| 141 | French fries | Fast food chains | None | Different brands | 9 | van Langeveld | 4 |
| 142 | Ice cream | Fast food chains | None | Different brands | 46 | van Langeveld | 4 |
| 143 | Italian hot dog | Fast food chains | None | Without brand | 10.3 | Martin | 1 |
| 144 | Pizza | Fast food chains | Pepperoni | Different brands | 16 | van Langeveld | 3 |
| 145 | Sandwich | Fast food chains | Olive tomato lettuce | Subway | 10.3 | Martin | 1 |
| 146 | Yoghurt | Fast food chains | None | MC Donald's | 30 | van Langeveld | 1 |
| 147 | Beef burgers | Meat and substitutes | None | Different brands | 6 | van Langeveld | 4 |
| 148 | Beef, different preparations | Meat and substitutes | None | Without brand | 6 | van Langeveld | 30 |
| 149 | Black sausage | Meat and substitutes | None | Without brand | 6 | van Langeveld | 1 |
| 150 | Chicken burger | Meat and substitutes | None | Different brands | 7 | van Langeveld | 3 |
| 151 | Chicken Nuggets | Meat and substitutes | None | Agrosuper | 7 | van Langeveld | 2 |
| 152 | Chicken, different preparations | Meat and substitutes | None | Without brand | 6 | van Langeveld | 27 |

| | | | | | | | |
|---|---|---|---|---|---|---|---|
| 153 | Ham, different types | Meat and substitutes | None | Different brands | 5 | van Langeveld | 35 |
| 154 | Mortadella, different types | Meat and substitutes | None | Different brands | 5 | van Langeveld | 2 |
| 155 | Pate, different types | Meat and substitutes | None | San Jorge | 15 | van Langeveld | 10 |
| 156 | Pepperoni | Meat and substitutes | None | Without brand | 5 | van Langeveld | 1 |
| 157 | Pork chop and rib, different preparations | Meat and substitutes | None | Without brand | 4 | van Langeveld | 5 |
| 158 | Pork sausage | Meat and substitutes | None | Without brand | 6 | van Langeveld | 2 |
| 159 | Pork steak and pulp, different preparations | Meat and substitutes | None | Without brand | 6 | van Langeveld | 4 |
| 160 | Pork, cooked loin | Meat and substitutes | None | Without brand | 6 | van Langeveld | 1 |
| 161 | Salami | Meat and substitutes | None | Different brands | 5 | van Langeveld | 5 |
| 162 | Turkey, breast and short ham | Meat and substitutes | None | Without brand | 6 | van Langeveld | 4 |
| 163 | Viennese, chicken | Meat and substitutes | None | Different brands | 3 | van Langeveld | 6 |
| 164 | Viennese, pork | Meat and substitutes | Pork | Different brands | 5 | van Langeveld | 10 |
| 165 | Viennese, turkey | Meat and substitutes | None | Different brands | 3 | van Langeveld | 5 |
| 166 | Alfajor | Grains and bread | None | Different brands | 59 | van Langeveld | 5 |
| 167 | Baked pie | Grains and bread | Pine tree | Without brand | 4 | van Langeveld | 1 |
| 168 | Baked pie | Grains and bread | Meat and cheese | Without brand | 4 | van Langeveld | 1 |
| 169 | Berlin with filling | Grains and bread | Different flavors | Without brand | 61 | van Langeveld | 2 |
| 170 | Bread, ciabatta | Grains and bread | None | Without brand | 6 | van Langeveld | 1 |
| 171 | Bread, coliza | Grains and bread | None | Without brand | 5 | van Langeveld | 1 |
| 172 | Bread, crumbs | Grains and bread | None | Without brand | 5 | van Langeveld | 1 |
| 173 | Bread, dobladita | Grains and bread | None | Without brand | 5 | van Langeveld | 1 |
| 174 | Bread, Duo whole grain | Grains and bread | None | Brown | 4 | van Langeveld | 1 |
| 175 | Bread, hallulla | Grains and bread | None | Without brand | 5 | van Langeveld | 2 |
| 176 | Bread, hamburger and, hot dog | Grains and bread | None | Different brands | 5 | van Langeveld | 2 |
| 177 | Bread, homemade | Grains and bread | None | Without brand | 5 | van Langeveld | 1 |
| 178 | Bread, marraqueta | Grains and bread | None | Without brand | 5 | van Langeveld | 1 |
| 179 | Bread, pita | Grains and bread | None | Ideal | 7 | van Langeveld | 1 |
| 180 | | Grains and bread | None | Without brand | 5 | van Langeveld | 1 |
| 181 | Bread, tapadito | Grains and bread | None | Without brand | 5 | van Langeveld | 1 |

| | | | | | | | |
|---|---|---|---|---|---|---|---|
| 182 | Bread, white | Grains and bread | None | Different brands | 5 | van Langeveld | 7 |
| 183 | Bread, whole grain | Grains and bread | None | Different brands | 4 | van Langeveld | 9 |
| 184 | Breakfast cereal, Check | Grains and bread | Yogurt, 3 cereals | Alive | 34 | van Langeveld | 2 |
| 185 | Breakfast cereal, Chocolate | Grains and bread | Chocolate | Different brands | 41 | van Langeveld | 16 |
| 186 | Breakfast cereal, Fruity Aritos | Grains and bread | None | Saint Elizabeth | 34 | van Langeveld | 1 |
| 187 | Breakfast cereal, sugary cereal | Grains and bread | None | Lider | 34 | van Langeveld | 1 |
| 188 | Breakfast cereal, without chocolate | Grains and bread | Different flavors | Different brands | 34 | van Langeveld | 20 |
| 189 | Cachito cake with caramel “manjar” | Grains and bread | None | Without brand | 61 | van Langeveld | 1 |
| 190 | Cake | Grains and bread | Vanilla | Different brands | 38 | van Langeveld | 8 |
| 191 | Cake | Grains and bread | Lemon | Without brand | 52 | van Langeveld | 1 |
| 192 | Cake, chocolate chip | Grains and bread | Chocolate chips | Without brand | 44 | van Langeveld | 1 |
| 193 | Cake, kequito | Grains and bread | Vanilla | Ideal | 38 | van Langeveld | 1 |
| 194 | Cake, magdalena | Grains and bread | Vanilla | Lider | 38 | van Langeveld | 1 |
| 195 | Cake, magdalena | Grains and bread | Vanilla | Ideal | 38 | van Langeveld | 1 |
| 196 | Cake, magdalena | Grains and bread | Vanilla with chocolate chips | Jumbo | 61 | van Langeveld | 1 |
| 197 | Cake, mankeke | Grains and bread | None | Marinela | 61 | van Langeveld | 1 |
| 198 | | Grains and bread | Pineapple | Jumbo | 61 | van Langeveld | 1 |
| 199 | Cake, Raspberry meringue | Grains and bread | Raspberry meringue | Without brand | 61 | van Langeveld | 1 |
| 200 | Cake, roll | Grains and bread | None | Marinela | 61 | van Langeveld | 1 |
| 201 | Cake, three milk | Grains and bread | Three milks | Lider | 61 | van Langeveld | 1 |
| 202 | Cake, yoghurt | Grains and bread | Yogurt | Different brands | 38 | van Langeveld | 2 |
| 203 | Cornmeal | Grains and bread | None | Without brand | 3 | van Langeveld | 1 |
| 204 | Cornstarch | Grains and bread | None | Without brand | 3 | van Langeveld | 1 |
| 205 | Cous cous | Grains and bread | None | Without brand | 3 | van Langeveld | 1 |
| 206 | Cracker with salt | Grains and bread | None | Different brands | 3 | van Langeveld | 6 |
| 207 | Cracker, Rice-with salt | Grains and bread | None | Different brands | 3 | van Langeveld | 3 |

| | | | | | | | |
|---|---|---|---|---|---|---|---|
| 208 | Cracker, Savory water | Grains and bread | None | Different brands | 3 | van Langeveld | 3 |
| 209 | Cracker, Selz mini with salt | Grains and bread | Different flavors | Arcor | 3 | van Langeveld | 3 |
| 210 | Cracker, Soda with salt | Grains and bread | None | Mckay | 3 | van Langeveld | 5 |
| 211 | Cuchuflí and Cuchufli stuffed | Grains and bread | None | Without brand | 61 | van Langeveld | 3 |
| 212 | Cupcake | Grains and bread | None | Without brand | 52 | van Langeveld | 1 |
| 213 | Fried pie | Grains and bread | Cheese | Without brand | 16 | van Langeveld | 1 |
| 214 | Grits | Grains and bread | None | Different brands | 3 | van Langeveld | 2 |
| 215 | Lemon pie | Grains and bread | None | Without brand | 54 | van Langeveld | 1 |
| 216 | Light soda cracker with salt | Grains and bread | None | Different brands | 3 | van Langeveld | 2 |
| 217 | Medialuna cake with caramel “manjar” | Grains and bread | Carmel “manjar” | Without brand | 61 | van Langeveld | 1 |
| 218 | Oat-based cereal | Grains and bread | None | Quaker | 34 | van Langeveld | 1 |
| 219 | Oatmeal / Instant Oatmeal | Grains and bread | None | Different brands | 14 | van Langeveld | 7 |
| 220 | Pasta, different types | Grains and bread | None | Different brands | 3 | van Langeveld | 47 |
| 221 | Pasta, ravioli | Grains and bread | None | Different brands | 8 | van Langeveld | 3 |
| 222 | Pizza | Grains and bread | Cheese and tomato sauce | Without brand | 16 | van Langeveld | 1 |
| 223 | Pizza mass | Grains and bread | None | Without brand | 11 | van Langeveld | 1 |
| 224 | Quinoa | Grains and bread | None | Different brands | 3 | van Langeveld | 2 |
| 225 | Rice | Grains and bread | None | Different brands | 3 | van Langeveld | 11 |
| 226 | Sopaipillas | Grains and bread | None | Without brand | 4 | van Langeveld | 1 |
| 227 | Sponge cake with filling | Grains and bread | Different flavors | Without brand | 61 | van Langeveld | 4 |
| 228 | Strudel | Grains and bread | Different flavors | Different brands | 61 | van Langeveld | 4 |
| 229 | Stuffed waffle cookie | Grains and bread | Peanut butter | Coast | 35 | van Langeveld | 1 |
| 230 | Sweet bread | Grains and bread | None | Different brands | 51 | van Langeveld | 5 |
| 231 | Sweet cookies with chocolate | Grains and bread | Chocolate | Different brands | 35 | van Langeveld | 32 |

| | | | | | | | |
|---|---|---|---|---|---|---|---|
| 232 | Sweet cookies without chocolate | Grains and bread | Different flavors | Different brands | 27 | van Langeveld | 63 |
| 233 | Unsalted soda cracker | Grains and bread | None | Without brand | 3 | van Langeveld | 1 |
| 234 | Wheat biscuit with bran and salt | Grains and bread | Wheat bran | Different brands | 3 | van Langeveld | 2 |
| 235 | Wheat flour | Grains and bread | None | Different brands | 3 | van Langeveld | 2 |
| 236 | Whole grain taco | Grains and bread | None | Without brand | 11 | van Langeveld | 1 |
| 237 | Apple, cooked | Fruits | None | Without brand | 22 | van Langeveld | 1 |
| 238 | Apple, Fuji | Fruits | None | Without brand | 20 | van Langeveld | 5 |
| 239 | Apple, green | Fruits | None | Without brand | 30 | van Langeveld | 6 |
| 240 | Apple, red | Fruits | None | Without brand | 20 | van Langeveld | 7 |
| 241 | Apricot | Fruits | None | Without brand | 30 | van Langeveld | 1 |
| 242 | Banana, green cooked | Fruits | Banana | Without brand | 3.7 | Martin | 1 |
| 243 | Blackberry | Fruits | None | Without brand | 30 | van Langeveld | 1 |
| 244 | Blonde raisins | Fruits | None | Without brand | 51 | van Langeveld | 1 |
| 245 | Blueberry | Fruits | None | Without brand | 30 | van Langeveld | 1 |
| 246 | Cherry | Fruits | None | Without brand | 30 | van Langeveld | 3 |
| 247 | Clementine | Fruits | None | Without brand | 30 | van Langeveld | 1 |
| 248 | Cucumber fruit | Fruits | None | Without brand | 30 | van Langeveld | 1 |
| 249 | Custard apple | Fruits | None | Without brand | 30 | van Langeveld | 1 |
| 250 | Dried plum | Fruits | None | Without brand | 51 | van Langeveld | 1 |
| 251 | Fruit compote with sugar | Fruits | Apple | Different brands | 37 | Sensory panel | 8 |
| 252 | Fruit compote with sugar | Fruits | Peach | Different brands | 37 | Sensory panel | 6 |
| 253 | Fruit compote with sugar | Fruits | Pear | Different brands | 37 | Sensory panel | 6 |
| 254 | Fruit compote with sugar | Fruits | Apple + sweet potato + carrot | Smiley Kids | 37 | Sensory panel | 1 |
| 255 | Fruit compote with sugar | Fruits | Apple with oatmeal | El Vergel | 37 | Sensory panel | 1 |
| 256 | Fruit compote without sugar | Fruits | Mango passion fruit apple | Vivo | 37 | Sensory panel | 1 |
| 257 | Fruit compote without sugar | Fruits | Mango | Regimel | 37 | Sensory panel | 1 |
| 258 | Fruit compote without sugar | Fruits | Apple and strawberry | Vivo | 37 | Sensory panel | 1 |

| | | | | | | | |
|---|---|---|---|---|---|---|---|
| 259 | Fruit compote without sugar | Fruits | Apple | Different brands | 37 | Sensory panel | 7 |
| 260 | Fruit compote without sugar | Fruits | Banana apple and orange | Vivo | 37 | Sensory panel | 1 |
| 261 | Fruit compote without sugar | Fruits | Pear + Banana + Mango | Smiley Kids | 37 | Sensory panel | 1 |
| 262 | Fruit compote without sugar | Fruits | Apple pear | Different brands | 37 | Sensory panel | 4 |
| 263 | Fruit compote without sugar | Fruits | Apple and peach | Dos Caballos | 37 | Sensory panel | 1 |
| 264 | Fruit compote without sugar | Fruits | Pear | Vivo | 37 | Sensory panel | 1 |
| 265 | Fruit, canned | Fruits | Peach pear cherry | El Vergel | 37 | van Langeveld | 1 |
| 266 | Grape | Fruits | None | Without brand | 30 | van Langeveld | 1 |
| 267 | Grape, green | Fruits | None | Without brand | 30 | van Langeveld | 1 |
| 268 | Grape, pink | Fruits | None | Without brand | 30 | van Langeveld | 1 |
| 269 | Grape, white | Fruits | None | Without brand | 30 | van Langeveld | 1 |
| 270 | Kiwi, cooked | Fruits | None | Without brand | 30 | van Langeveld | 1 |
| 271 | Kiwi | Fruits | None | Without brand | 19 | van Langeveld | 1 |
| 272 | Lemon, pica | Fruits | None | Without brand | 3.7 | Martin | 1 |
| 273 | Lemon, fruit, juice, peel | Fruits | None | Without brand | 3.7 | Martin | 1 |
| 274 | Mango | Fruits | None | Without brand | 30 | van Langeveld | 2 |
| 275 | Mango, cooked | Fruits | None | Without brand | 30 | van Langeveld | 1 |
| 276 | Maqui powder | Fruits | None | Without brand | 51 | van Langeveld | 1 |
| 277 | Melon | Fruits | None | Without brand | 30 | van Langeveld | 1 |
| 278 | Melon, calameño | Fruits | None | Without brand | 30 | van Langeveld | 1 |
| 279 | Melon, tuna | Fruits | None | Without brand | 30 | van Langeveld | 1 |
| 280 | Orange | Fruits | None | Without brand | 19 | van Langeveld | 2 |
| 281 | Orange peel | Fruits | None | Without brand | 3.7 | Martin | 1 |
| 282 | Peach, conservero | Fruits | None | Without brand | 30 | van Langeveld | 1 |
| 283 | Peach, conservero cooked | Fruits | None | Without brand | 30 | van Langeveld | 1 |
| 284 | Peach, banana | Fruits | None | Without brand | 30 | van Langeveld | 1 |
| 285 | Peach, canned | Fruits | None | Dos Caballos | 31 | van Langeveld | 1 |
| 286 | Peach, nectarine | Fruits | None | Without brand | 30 | van Langeveld | 2 |
| 287 | Peach, nectarine cooked | Fruits | None | Without brand | 30 | van Langeveld | 1 |
| 288 | Pear | Fruits | None | Without brand | 30 | van Langeveld | 5 |
| 289 | Pear, cooked | Fruits | None | Without brand | 30 | van Langeveld | 1 |
| 290 | Pineapple | Fruits | None | Without brand | 34 | van Langeveld | 1 |

| | | | | | | | |
|---|---|---|---|---|---|---|---|
| 291 | Pineapple, canned | Fruits | None | Without brand | 37 | van Langeveld | 1 |
| 292 | Plantain | Fruits | None | Without brand | 29 | van Langeveld | 1 |
| 293 | Plum | Fruits | None | Without brand | 30 | van Langeveld | 2 |
| 294 | Quince, cooked | Fruits | None | Without brand | 30 | van Langeveld | 1 |
| 295 | Raisins | Fruits | None | Without brand | 51 | van Langeveld | 1 |
| 296 | Raspberry | Fruits | Raspberry | Minuto verde | 18 | van Langeveld | 1 |
| 297 | Strawberries, canned | Fruits | None | Without brand | 37 | van Langeveld | 1 |
| 298 | Strawberry | Fruits | None | Without brand | 18 | van Langeveld | 1 |
| 299 | Strawberry, cooked | Fruits | None | Without brand | 30 | van Langeveld | 1 |
| 300 | Tangerine | Fruits | None | Without brand | 26 | van Langeveld | 1 |
| 301 | Watermelon | Fruits | None | Without brand | 30 | van Langeveld | 1 |
| 302 | Almond | Fats and Oils | None | Without brand | 13 | van Langeveld | 1 |
| 303 | Avocado | Fats and Oils | None | Without brand | 3 | van Langeveld | 1 |
| 304 | Butter | Fats and Oils | None | Different brands | 3 | van Langeveld | 5 |
| 305 | Butter, canola oil spread | Fats and Oils | None | Calo | 3 | van Langeveld | 1 |
| 306 | Butter, light | Fats and Oils | None | Calo | 3 | van Langeveld | 1 |
| 307 | Butter, spreadable | Fats and Oils | None | Soprole | 3 | van Langeveld | 1 |
| 308 | Butter, unsalted | Fats and Oils | None | Different brands | 3 | van Langeveld | 3 |
| 309 | Chia | Fats and Oils | None | Without brand | 13 | van Langeveld | 1 |
| 310 | Hazelnut, roasted | Fats and Oils | None | Without brand | 13 | van Langeveld | 1 |
| 311 | Lard | Fats and Oils | None | Without brand | 5 | van Langeveld | 1 |
| 312 | Margarine | Fats and Oils | None | Different brands | 3 | van Langeveld | 6 |
| 313 | Margarine with butter, Qualy | Fats and Oils | None | Sadia | 3 | van Langeveld | 1 |
| 314 | Margarine, Creamy Qualy | Fats and Oils | None | Sadia | 5 | van Langeveld | 1 |
| 315 | Margarine, light | Fats and Oils | Butter | Different brands | 2 | van Langeveld | 8 |
| 316 | Margarine, mix with butter | Fats and Oils | None | Soprole | 3 | van Langeveld | 1 |
| 317 | Margarine, Next | Fats and Oils | None | Soprole | 3 | van Langeveld | 1 |
| 318 | Nut | Fats and Oils | None | Without brand | 13 | van Langeveld | 1 |
| 319 | Oil, canola | Fats and Oils | None | Different brands | 2 | van Langeveld | 4 |
| 320 | Oil, chia | Fats and Oils | None | Without brand | 2 | van Langeveld | 1 |
| 321 | Oil, grapeseed | Fats and Oils | None | Different brands | 2 | van Langeveld | 3 |
| 322 | Oil, olive | Fats and Oils | None | Different brands | 1 | van Langeveld | 14 |

| | | | | | | | |
|---|---|---|---|---|---|---|---|
| 323 | Oil, sunflower | Fats and Oils | None | Different brands | 2 | van Langeveld | 11 |
| 324 | Oil, vegetable | Fats and Oils | None | Different brands | 2 | van Langeveld | 11 |
| 325 | Peanut | Fats and Oils | None | Different brands | 8 | van Langeveld | 3 |
| 326 | Artichoke | Vegetables / algae and mushrooms | None | Without brand | 9 | van Langeveld | 2 |
| 327 | Asparagus | Vegetables / algae and mushrooms | None | Without brand | 9 | van Langeveld | 2 |
| 328 | Basil | Vegetables / algae and mushrooms | None | Without brand | 9 | van Langeveld | 1 |
| 329 | Beetroot, cooked | Vegetables / algae and mushrooms | None | Without brand | 9 | van Langeveld | 2 |
| 330 | Broccoli | Vegetables / algae and mushrooms | None | Without brand | 6 | van Langeveld | 1 |
| 331 | Broccoli, cooked | Vegetables / algae and mushrooms | None | Without brand | 6 | van Langeveld | 1 |
| 332 | Cabbage, brussels | Vegetables / algae and mushrooms | None | Without brand | 8 | van Langeveld | 1 |
| 333 | Cabbage, green | Vegetables / algae and mushrooms | None | Without brand | 9 | van Langeveld | 1 |
| 334 | Cabbage, purple | Vegetables / algae and mushrooms | None | Without brand | 9 | van Langeveld | 1 |
| 335 | Carrot | Vegetables / algae and mushrooms | None | Without brand | 15 | van Langeveld | 7 |
| 336 | Cauliflower | Vegetables / algae and mushrooms | None | Without brand | 6 | van Langeveld | 1 |
| 337 | Celery | Vegetables / algae and mushrooms | None | Without brand | 12 | van Langeveld | 2 |
| 338 | Chard | Vegetables / algae and mushrooms | None | Without brand | 9 | van Langeveld | 2 |
| 339 | Chives | Vegetables / algae and mushrooms | None | Without brand | 9 | van Langeveld | 2 |
| 340 | Chives | Vegetables / algae and mushrooms | None | Without brand | 9 | van Langeveld | 1 |
| 341 | Coriander | Vegetables / algae and mushrooms | None | Without brand | 9 | van Langeveld | 1 |
| 342 | Corn | Vegetables / algae and mushrooms | None | Without brand | 9 | van Langeveld | 3 |

| | | | | | | | |
|---|---|---|---|---|---|---|---|
| 343 | Corn kernels | Vegetables / algae and mushrooms | None | Different brands | 9 | van Langeveld | 5 |
| 344 | Cucumber salad | Vegetables / algae and mushrooms | None | Without brand | 6 | van Langeveld | 1 |
| 345 | Dragon tooth | Vegetables / algae and mushrooms | None | Without brand | 8 | van Langeveld | 1 |
| 346 | Dried oregano | Vegetables / algae and mushrooms | None | Different brands | 9 | van Langeveld | 3 |
| 347 | Eggplant cooked | Vegetables / algae and mushrooms | None | Without brand | 9 | van Langeveld | 1 |
| 348 | Frozen fries | Vegetables / algae and mushrooms | None | Without brand | 9 | van Langeveld | 1 |
| 349 | Garlic | Vegetables / algae and mushrooms | None | Without brand | 9 | van Langeveld | 1 |
| 350 | Garlic cooked | Vegetables / algae and mushrooms | None | Without brand | 9 | van Langeveld | 1 |
| 351 | Green beans | Vegetables / algae and mushrooms | None | Without brand | 9 | van Langeveld | 2 |
| 352 | Instant Pot Mashed | Vegetables / algae and mushrooms | None | Without brand | 7 | van Langeveld | 1 |
| 353 | Laurel | Vegetables / algae and mushrooms | None | Without brand | 9 | van Langeveld | 1 |
| 354 | Lettuce | Vegetables / algae and mushrooms | None | Without brand | 9 | van Langeveld | 1 |
| 355 | Mushroom | Vegetables / algae and mushrooms | None | Without brand | 7 | van Langeveld | 1 |
| 356 | Mushroom canned | Vegetables / algae and mushrooms | None | Without brand | 7 | van Langeveld | 1 |
| 357 | Nori seaweed | Vegetables / algae and mushrooms | None | Without brand | 9 | van Langeveld | 1 |
| 358 | Olives, black | Vegetables / algae and mushrooms | None | Without brand | 4 | van Langeveld | 1 |
| 359 | Olives, green | Vegetables / algae and mushrooms | None | Without brand | 4 | van Langeveld | 1 |
| 360 | Onion | Vegetables / algae and mushrooms | None | Without brand | 7 | van Langeveld | 3 |
| 361 | Onion, pickled | Vegetables / algae and mushrooms | None | Without brand | 10 | van Langeveld | 1 |
| 362 | Onion, purple | Vegetables / algae and mushrooms | None | Without brand | 7 | van Langeveld | 1 |

| | | | | | | | |
|---|---|---|---|---|---|---|---|
| 363 | Oregano | Vegetables / algae and mushrooms | None | Without brand | 9 | van Langeveld | 1 |
| 364 | Paprika, green | Vegetables / algae and mushrooms | None | Without brand | 7 | van Langeveld | 2 |
| 365 | Paprika, red | Vegetables / algae and mushrooms | None | Without brand | 14 | van Langeveld | 2 |
| 366 | Paprika, yellow | Vegetables / algae and mushrooms | None | Without brand | 16 | van Langeveld | 1 |
| 367 | Parsley | Vegetables / algae and mushrooms | None | Without brand | 9 | van Langeveld | 1 |
| 368 | Pickles | Vegetables / algae and mushrooms | None | Without brand | 9 | van Langeveld | 1 |
| 369 | Potato, duchess | Vegetables / algae and mushrooms | None | Without brand | 7 | van Langeveld | 1 |
| 370 | Potato, pre-fried | Vegetables / algae and mushrooms | None | Without brand | 9 | van Langeveld | 2 |
| 371 | Potato, white | Vegetables / algae and mushrooms | None | Without brand | 5 | van Langeveld | 3 |
| 372 | Potato, yellow | Vegetables / algae and mushrooms | None | Without brand | 5 | van Langeveld | 1 |
| 373 | Spinach | Vegetables / algae and mushrooms | None | Without brand | 7 | van Langeveld | 3 |
| 374 | Spring vegetables | Vegetables / algae and mushrooms | None | Minuto Verde | 9 | van Langeveld | 1 |
| 375 | Squash | Vegetables / algae and mushrooms | None | Without brand | 9 | van Langeveld | 1 |
| 376 | Squash, italian | Vegetables / algae and mushrooms | None | Without brand | 9 | van Langeveld | 3 |
| 377 | Squash, sweet potato | Vegetables / algae and mushrooms | None | Without brand | 9 | van Langeveld | 2 |
| 378 | Tomato | Vegetables / algae and mushrooms | None | Without brand | 10 | van Langeveld | 5 |
| 379 | Tomato, cherry | Vegetables / algae and mushrooms | None | Without brand | 10 | van Langeveld | 1 |
| 380 | Yucca | Vegetables / algae and mushrooms | None | Without brand | 9 | van Langeveld | 1 |
| 381 | Egg, white, yolk, boiled, fried, scrambled and others | Eggs and egg products | None | Without brand | 5 | van Langeveld | 10 |
| 382 | Almond drink | Dairy and substitutes | Almond | Without brand | 30 | Sensory panel | 2 |
| 383 | Rice drink | Dairy and substitutes | None | Osari | 30 | Sensory panel | 1 |

| | | | | | | | |
|---|---|---|---|---|---|---|---|
| 384 | Coconut drink | Dairy and substitutes | Coconut | Dream | 30 | Sensory panel | 1 |
| 385 | Soy drink | Dairy and substitutes | Different flavors | Different brands | 30 | Sensory panel | 5 |
| 386 | Milk drink, powdered whole | Dairy and substitutes | Chocolate | Macro Food | 37 | van Langeveld | 1 |
| 387 | Milk drink, Milo | Dairy and substitutes | Chocolate | Nestle | 37 | van Langeveld | 1 |
| 388 | Milk drink, Mono choc | Dairy and substitutes | Chocolate | Costa | 37 | van Langeveld | 1 |
| 389 | Milk drink, Chamyto probiotic | Dairy and substitutes | Different flavors | Nestle | 39 | van Langeveld | 5 |
| 390 | Milk drink, Chiquitin surprise probiotic | Dairy and substitutes | None | Nestle | 39 | van Langeveld | 1 |
| 391 | Milk drink, Calan Multifruit Probiotic | Dairy and substitutes | Multi-fruit | Danone | 50 | van Langeveld | 1 |
| 392 | Milk drink, Probiotic One a day | Dairy and substitutes | Different flavors | Soprole | 39 | van Langeveld | 3 |
| 393 | Milk drink, Yogu yogu | Dairy and substitutes | Strawberry | Loncoleche | 39 | van Langeveld | 1 |
| 394 | Cream, Chantilly | Dairy and substitutes | None | Different brands | 49 | van Langeveld | 2 |
| 395 | Cream Milk | Dairy and substitutes | None | Different brands | 49 | van Langeveld | 2 |
| 396 | Cream Thick | Dairy and substitutes | None | Soprole | 49 | van Langeveld | 1 |
| 397 | Milk buckets | Dairy and substitutes | Plantain | Without brand | 46 | van Langeveld | 1 |
| 398 | Ice Cream, milk-based | Dairy and substitutes | Different flavors | Different brands | 48 | van Langeveld | 32 |
| 399 | Milk, condensed | Dairy and substitutes | None | Without brand | 88 | van Langeveld | 1 |
| 400 | Milk, cultured diet unsweetened | Dairy and substitutes | Strawberry | Parmalat | 39 | van Langeveld | 1 |
| 401 | Milk, cultured without sugar cultured | Dairy and substitutes | Custard apple | Soprole | 39 | van Langeveld | 1 |
| 402 | Milk, goat | Dairy and substitutes | Goat milk | Praderia | 39 | van Langeveld | 1 |
| 403 | Milk, skim | Dairy and substitutes | None | Different brands | 14 | van Langeveld | 1 |
| 404 | Milk, skim | Dairy and substitutes | Chocolate | Soprole | 39 | van Langeveld | 1 |
| 405 | Milk, light skim | Dairy and substitutes | Chocolate | Soprole | 39 | van Langeveld | 1 |
| 406 | Milk, light skim | Dairy and substitutes | Chocolate | Loncoleche | 39 | van Langeveld | 1 |
| 407 | Milk, lactose-free skim | Dairy and substitutes | None | Soprole | 14 | van Langeveld | 1 |
| 408 | Milk, lactose-free skim Slim | Dairy and substitutes | Chocolate | Different brands | 39 | van Langeveld | 2 |
| 409 | Milk, whole | Dairy and substitutes | None | Different brands | 12 | Sensory panel | 5 |
| 410 | Milk, whole powder | Dairy and substitutes | None | Different brands | 12 | Sensory panel | 5 |
| 411 | Milk, lactose-free whole | Dairy and substitutes | None | Colun | 12 | Sensory panel | 1 |

| | | | | | | | |
|---|---|---|---|---|---|---|---|
| 412 | Milk, evaporated | Dairy and substitutes | None | Gloria | 39 | van Langeveld | 1 |
| 413 | Milk, low-fat | Dairy and substitutes | None | Different brands | 12 | Sensory panel | 6 |
| 414 | Milk, low-fat with sugar | Dairy and substitutes | Different flavors | Different brands | 39 | van Langeveld | 13 |
| 415 | Milk, low-fat with sugar Nido | Dairy and substitutes | Chocolate | Nestle | 39 | van Langeveld | 1 |
| 416 | Milk, semi-skimmed with sugar Surlat Kids | Dairy and substitutes | Chocolate | Surlat | 39 | van Langeveld | 1 |
| 417 | Milk, semi-skimmed powder | Dairy and substitutes | None | Different brands | 12 | Sensory panel | 3 |
| 418 | Milk, low-fat powder with sugar | Dairy and substitutes | Different flavors | JUNJI | 37 | Sensory panel | 5 |
| 419 | Milk, semi-skimmed powder Crecer 1+ | Dairy and substitutes | None | Calo | 12 | Sensory panel | 1 |
| 420 | Milk, semi-skimmed powder Crecer 4+ | Dairy and substitutes | None | Calo | 12 | Sensory panel | 1 |
| 421 | Milk, semi-skimmed powder | Dairy and substitutes | natural | JUNAEB | 12 | Sensory panel | 1 |
| 422 | Milk, semi-skimmed powder Different Stages | Dairy and substitutes | None | Nestle | 12 | Sensory panel | 5 |
| 423 | Milk, protein + semi-skimmed | Dairy and substitutes | Chocolate | Soprole | 39 | van Langeveld | 1 |
| 424 | Milk, lactose-free semi-skim | Dairy and substitutes | None | Different brands | 12 | Sensory panel | 3 |
| 425 | Dessert, Chiquitín | Dairy and substitutes | Different flavors | Nestle | 30 | van Langeveld | 1 |
| 426 | Dessert, flan | Dairy and substitutes | Vanilla | Different brands | 33 | van Langeveld | 4 |
| 427 | Dessert, flan | Dairy and substitutes | Candy | Different brands | 36 | van Langeveld | 3 |
| 428 | Dessert, Flan with caramel sauce | Dairy and substitutes | Delicacy | Colun | 34 | van Langeveld | 1 |
| 429 | Dessert, Flan with caramel sauce | Dairy and substitutes | Chocolate | Colun | 36 | van Langeveld | 1 |
| 430 | Dessert, Roasted milk | Dairy and substitutes | None | Nestle | 43 | van Langeveld | 1 |
| 431 | Dessert, Manjarate | Dairy and substitutes | Delicacy | Soprole | 46 | van Langeveld | 1 |
| 432 | Dessert, Semolina with milk | Dairy and substitutes | Caramel sauce | Soprole | 43 | van Langeveld | 1 |
| 433 | Quesillo | Dairy and substitutes | None | Different brands | 5 | van Langeveld | 4 |
| 434 | Cheese, chacra | Dairy and substitutes | None | Quillayes | 6 | van Langeveld | 1 |
| 435 | Cheese, chanco | Dairy and substitutes | None | Without brand | 6 | van Langeveld | 1 |
| 436 | Cheese, cheddar | Dairy and substitutes | None | Without brand | 6 | van Langeveld | 1 |
| 437 | Cheese, cream | Dairy and substitutes | Basil garlic | Colun | 8 | van Langeveld | 1 |
| 438 | Cheese, goat | Dairy and substitutes | None | Without brand | 9 | van Langeveld | 1 |
| 439 | Cheese, fresh | Dairy and substitutes | None | Different brands | 5 | van Langeveld | 3 |

| | | | | | | | |
|---|---|---|---|---|---|---|---|
| 440 | Cheese, gouda | Dairy and substitutes | None | Different brands | 6 | van Langeveld | 7 |
| 441 | Cheese, granulated | Dairy and substitutes | None | Without brand | 6 | van Langeveld | 1 |
| 442 | Cheese, gruyere | Dairy and substitutes | None | Without brand | 6 | van Langeveld | 1 |
| 443 | Cheese, laminated | Dairy and substitutes | None | Without brand | 6 | van Langeveld | 1 |
| 444 | Cheese, buttery | Dairy and substitutes | None | Different brands | 6 | van Langeveld | 4 |
| 445 | Cheese, grated parmesan | Dairy and substitutes | None | Lider | 6 | van Langeveld | 1 |
| 446 | Cheese, grated | Dairy and substitutes | None | Without brand | 6 | van Langeveld | 1 |
| 447 | Cheese, ranco | Dairy and substitutes | None | Different brands | 6 | van Langeveld | 2 |
| 448 | Cheese, grated reggianito | Dairy and substitutes | None | Colun | 6 | van Langeveld | 1 |
| 449 | Yoghurt, natural smoothie without sugar | Dairy and substitutes | natural | Colun | 2 | van Langeveld | 1 |
| 450 | Yoghurt, Low-fat shaken with sugar | Dairy and substitutes | Strawberry | Colun | 32 | van Langeveld | 14 |
| 451 | Yoghurt, Unsweetened nonfat smoothie | Dairy and substitutes | Strawberry | Surlat | 30 | van Langeveld | 1 |
| 452 | Yoghurt, whole-milk shaken with sugar | Dairy and substitutes | Different flavors | Different brands | 32 | van Langeveld | 34 |
| 453 | Yoghurt, whole-milk lactose-free smoothie with sugar | Dairy and substitutes | Different flavors | Different brands | 32 | van Langeveld | 3 |
| 454 | Yoghurt, low-fat smoothie with sugar | Dairy and substitutes | Different flavors | Different brands | 32 | van Langeveld | 16 |
| 455 | Yoghurt, semi-skimmed smoothie with sugar Surlat Kids | Dairy and substitutes | Strawberry | Surlat | 32 | van Langeveld | 1 |
| 456 | Yoghurt, semi-skimmed smoothie with sugar Surlat Kids | Dairy and substitutes | Blackberry | Surlat | 32 | van Langeveld | 1 |
| 457 | Yoghurt, Unsweetened low-fat smoothie | Dairy and substitutes | Strawberry | Soprole | 30 | van Langeveld | 1 |
| 458 | Soy Yogurt | Dairy and substitutes | peach | Loncoleche | 30 | van Langeveld | 1 |
| 459 | Yoghurt, Greek sugar skim | Dairy and substitutes | Strawberry | Colun | 42 | van Langeveld | 1 |
| 460 | Yoghurt, Low-fat with lactose- without sugar | Dairy and substitutes | Blackberry | Soprole | 42 | van Langeveld | 1 |
| 461 | Yoghurt, whole-milk Batifrut classic with sugar | Dairy and substitutes | peach | Soprole | 40 | van Langeveld | 1 |
| 462 | Yoghurt, whole-milk with sugar | Dairy and substitutes | None | Without brand | 40 | van Langeveld | 1 |
| 463 | Yoghurt, whole-milk with sugar Batifrut classic | Dairy and substitutes | Blackberry | Soprole | 40 | van Langeveld | 1 |
| 464 | Yoghurt, whole-milk with sugar Batifrut classic | Dairy and substitutes | Pineapple | Soprole | 40 | van Langeveld | 1 |
| 465 | Yoghurt, whole-milk with sugar Gold classic | Dairy and substitutes | Traditional | Soprole | 40 | van Langeveld | 1 |
| 466 | Yoghurt, whole-milk with Greek sugar | Dairy and substitutes | peach | Danone | 40 | van Langeveld | 1 |
| 467 | Yoghurt, whole-milk with Greek sugar | Dairy and substitutes | Strawberry | Danone | 40 | van Langeveld | 1 |

| | | | | | | | |
|---|---|---|---|---|---|---|---|
| 468 | Yoghurt, whole-milk with Milo sugar | Dairy and substitutes | Milo | Nestle | 40 | van Langeveld | 1 |
| 469 | Yoghurt, whole-milk with cereal with sugar | Dairy and substitutes | Different flavors | Different brands | 40 | van Langeveld | 3 |
| 470 | Yoghurt, whole-milk plain with sugar | Dairy and substitutes | None | Different brands | 30 | van Langeveld | 3 |
| 471 | Yoghurt, whole-milk plain unsweetened Greek | Dairy and substitutes | None | Danone | 2 | van Langeveld | 1 |
| 472 | Yoghurt, 1 + 1 lactose-free whole-milk Choco krispis with sugar | Dairy and substitutes | None | Soprole | 40 | van Langeveld | 1 |
| 473 | Yoghurt, Nonfat liquid with sugar and Probiotic | Dairy and substitutes | Strawberry | Calo | 32 | van Langeveld | 1 |
| 474 | Yoghurt, Low-fat liquid with sugar | Dairy and substitutes | Mango | Gloria | 32 | van Langeveld | 1 |
| 475 | Yoghurt, Unsweetened plain | Dairy and substitutes | natural | Regimel | 6 | van Langeveld | 1 |
| 476 | Yoghurt, low-fat with Greek sugar | Dairy and substitutes | peach | Quillayes | 40 | van Langeveld | 1 |
| 477 | Beans, broad | Legumes | None | Without brand | 13 | van Langeveld | 2 |
| 478 | Beans, white | Legumes | None | Without brand | 8 | van Langeveld | 3 |
| 479 | Chickpea | Legumes | None | Without brand | 13 | van Langeveld | 1 |
| 480 | Fresh peas | Legumes | None | Different brands | 15 | van Langeveld | 5 |
| 481 | Lentil | Legumes | None | Without brand | 13 | van Langeveld | 1 |
| 482 | Baking soda | Miscellaneous | None | Without brand | 10 | van Langeveld | 1 |
| 483 | Easter bread essence | Miscellaneous | None | Without brand | 50 | van Langeveld | 1 |
| 484 | Unflavored gelatin | Miscellaneous | None | Without brand | 0 | van Langeveld | 1 |
| 485 | Yeast | Miscellaneous | None | Without brand | 10 | van Langeveld | 1 |
| 486 | Baking powders | Miscellaneous | None | Without brand | 10 | van Langeveld | 1 |
| 487 | Nessucar supplement | Miscellaneous | None | Nestle | 54.5 | Sensory panel | 1 |
| 488 | Pediasure Supplement | Miscellaneous | Different flavors | Abbott | 54.5 | Sensory panel | 4 |
| 489 | Promise pe gold supplement | Miscellaneous | Vanilla | Wyeth | 54.5 | Sensory panel | 1 |
| 490 | Vanilla essence | Miscellaneous | None | Gourmet | 50 | van Langeveld | 1 |
| 491 | Albacore Cooked | Fish and shellfish | None | Without brand | 4 | van Langeveld | 1 |
| 492 | Tuna, Canned tuna in water | Fish and shellfish | None | Different brands | 4 | van Langeveld | 8 |
| 493 | Tuna, Canned Tuna Tenderloins in oil | Fish and shellfish | None | Different brands | 3 | van Langeveld | 3 |
| 494 | Shrimp, Cooked shrimp | Fish and shellfish | None | Without brand | 8 | van Langeveld | 1 |
| 495 | Malton mussel, baked | Fish and shellfish | None | Without brand | 4 | van Langeveld | 1 |
| 496 | Mussels with sunflower oil, canned | Fish and shellfish | None | Robinson Crusoe | 4 | van Langeveld | 1 |

| | | | | | | | |
|---|---|---|---|---|---|---|---|
| 497 | Hake dinos, baked | Fish and shellfish | None | El Golfo | 5 | van Langeveld | 1 |
| 498 | Cuttlefish, cooked | Fish and shellfish | None | Without brand | 4 | van Langeveld | 1 |
| 499 | Horse mackerel, canned in water | Fish and shellfish | None | Different brands | 4 | van Langeveld | 2 |
| 500 | Seafood Mash | Fish and shellfish | None | Without brand | 4 | van Langeveld | 1 |
| 501 | Hake, grilled | Fish and shellfish | None | Without brand | 4 | van Langeveld | 1 |
| 502 | Hake, cooked | Fish and shellfish | None | Without brand | 4 | van Langeveld | 2 |
| 503 | Hake, fried | Fish and shellfish | None | Without brand | 4 | van Langeveld | 1 |
| 504 | Fish, cooked | Fish and shellfish | None | Without brand | 4 | van Langeveld | 1 |
| 505 | Pomfret fish, roasted | Fish and shellfish | None | Without brand | 4 | van Langeveld | 1 |
| 506 | Pomfret, cooked | Fish and shellfish | None | Without brand | 4 | van Langeveld | 1 |
| 507 | Salmon, roasted | Fish and shellfish | None | Without brand | 4 | van Langeveld | 1 |
| 508 | Salmon, canned in water | Fish and shellfish | None | Without brand | 4 | van Langeveld | 1 |
| 509 | Black pepper | Sauces. condiments and dehydrated soups | None | Without brand | 0 | van Langeveld | 1 |
| 510 | Broth concentrate in tablet | Sauces. condiments and dehydrated soups | None | Without brand | 3.7 | Sensory panel | 1 |
| 511 | Burger base | Sauces. condiments and dehydrated soups | Burger | Without brand | 3.7 | Sensory panel | 1 |
| 512 | Chicken base, Maggi | Sauces. condiments and dehydrated soups | None | Nestle | 3.7 | Sensory panel | 1 |
| 513 | Chili pepper, color | Sauces. condiments and dehydrated soups | None | Without brand | 0 | van Langeveld | 1 |
| 514 | Cinnamon, ground and stick | Sauces. condiments and dehydrated soups | None | Without brand | 0 | van Langeveld | 2 |
| 515 | Clove | Sauces. condiments and dehydrated soups | None | Without brand | 0 | van Langeveld | 1 |
| 516 | Cream, reconstituted dehydrated | Sauces. condiments and dehydrated soups | Different flavors | Different brands | 3.7 | Sensory panel | 8 |
| 517 | Cumin | Sauces. condiments and dehydrated soups | None | Without brand | 0 | van Langeveld | 1 |
| 518 | Curry | Sauces. condiments and dehydrated soups | None | Without brand | 0 | van Langeveld | 1 |
| 519 | Dressing, complete | Sauces. condiments and dehydrated soups | None | Without brand | 0 | van Langeveld | 1 |
| 520 | Garlic powder | Sauces. condiments and dehydrated soups | None | Different brands | 0 | van Langeveld | 2 |
| 521 | Ketchup | Sauces. condiments and dehydrated soups | None | Different brands | 10 | van Langeveld | 13 |

| | | | | | | | |
|---|---|---|---|---|---|---|---|
| 522 | Mayonnaise | Sauces. condiments and dehydrated soups | None | Different brands | 10 | van Langeveld | 3 |
| 523 | Mayonnaise, light | Sauces. condiments and dehydrated soups | None | Lider | 10 | van Langeveld | 1 |
| 524 | Mustard seasoning | Sauces. condiments and dehydrated soups | None | Different brands | 6 | van Langeveld | 3 |
| 525 | Nutmeg | Sauces. condiments and dehydrated soups | None | Without brand | 0 | van Langeveld | 1 |
| 526 | Lemon juice substitute | Sauces. condiments and dehydrated soups | None | Traverso | 3.7 | Martin | 1 |
| 527 | Paprika powder | Sauces. condiments and dehydrated soups | None | Without brand | 0 | van Langeveld | 1 |
| 528 | Salt, Biosal reduced in sodium | Sauces. condiments and dehydrated soups | None | Biosal | 0 | van Langeveld | 1 |
| 529 | Salt, Cahuil | Sauces. condiments and dehydrated soups | None | Without brand | 0 | van Langeveld | 1 |
| 530 | Salt, coarse | Sauces. condiments and dehydrated soups | None | Different brands | 0 | van Langeveld | 2 |
| 531 | Salt, himalayan pink | Sauces. condiments and dehydrated soups | None | Manare | 0 | van Langeveld | 1 |
| 532 | Salt, fine | Sauces. condiments and dehydrated soups | None | Different brands | 0 | van Langeveld | 10 |
| 533 | Salt, light sodium | Sauces. condiments and dehydrated soups | None | Lobos | 0 | van Langeveld | 1 |
| 534 | Salt, potassium Biosal | Sauces. condiments and dehydrated soups | None | Biosal | 0 | van Langeveld | 1 |
| 535 | Salt, sea | Sauces. condiments and dehydrated soups | None | Different brands | 0 | van Langeveld | 1 |
| 536 | Soy sauce | Sauces. condiments and dehydrated soups | None | Without brand | 10 | van Langeveld | 1 |
| 537 | Soup, reconstituted dehydrated soup | Sauces. condiments and dehydrated soups | Different flavors | Different brands | 3.7 | Sensory panel | 4 |
| 538 | Tomato sauce, Natural Pomarola | Sauces. condiments and dehydrated soups | None | Carozzi | 10 | van Langeveld | 1 |
| 539 | Tomato sauce with meat, Tuco Maggi | Sauces. condiments and dehydrated soups | None | Nestle | 10 | van Langeveld | 1 |
| 540 | Vinegar, apple | Sauces. condiments and dehydrated soups | Apple | Traverso | 10 | van Langeveld | 1 |
| 541 | Vinegar, balsamic | Sauces. condiments and dehydrated soups | None | Different brands | 10 | van Langeveld | 2 |

| | | | | | | | |
|---|---|---|---|---|---|---|---|
| 542 | Vinegar, white | Sauces. condiments and dehydrated soups | None | Without brand | 10 | van Langeveld | 2 |
| 543 | Cereal bar with sugar | Snacks | Chocolate | Different brands | 18 | van Langeveld | 3 |
| 544 | Cereal bar with sugar | Snacks | Apple | Quaker | 35 | van Langeveld | 1 |
| 545 | Cereal bar without sugar | Snacks | Chocolate | En Línea | 45 | van Langeveld | 1 |
| 546 | Popcorn sweet | Snacks | None | Without brand | 43 | van Langeveld | 1 |
| 547 | Snack, savory Rustic French Fries | Snacks | Merkén | Marco Polo | 11 | van Langeveld | 1 |
| 548 | Snack, sweet dried apple chips | Snacks | None | Without brand | 35 | van Langeveld | 1 |
| 549 | Snack, sweet confited peanuts | Snacks | None | Without brand | 42 | van Langeveld | 1 |
| 550 | Snack, mix | Snacks | None | Evercrisp | 11 | van Langeveld | 1 |
| 551 | Snack, nuts mix | Snacks | None | Different brands | 12 | van Langeveld | 5 |
| 552 | Snack, savory Chanfles | Snacks | Cheese | Fruna | 12 | van Langeveld | 1 |
| 553 | Snack, savory Peanuts almonds cashews | Snacks | None | Marco Polo | 12 | van Langeveld | 1 |
| 554 | Snack, savory French fries | Snacks | Different flavors | Different brands | 11 | van Langeveld | 11 |
| 555 | Snack, savory Twigs | Snacks | Different flavors | Different brands | 12 | van Langeveld | 4 |
| 556 | Snack, savory Sufflé | Snacks | Different flavors | Different brands | 12 | van Langeveld | 10 |
| 557 | Snack, savory corn tortilla | Snacks | None | Different brands | 12 | van Langeveld | 1 |
| 558 | Snack, sweet Soufflé | Snacks | Different flavors | Different brands | 35 | van Langeveld | 1 |

* When more than one product is represented, the same sweetness intensity value was used to homologate a set of similar products. For this reason, the table displays sweetness intensity values for 558 products, which represents total products found in participants' dietary records at both ages (i.e., 1,737 products).
