# Supplementary material for: Sweetness of Chilean Infants’ Diets: Methodology and Description
Source: Nutrients. 2022 Mar 30;14(7):1447. doi: 10.3390/nu14071447 (PMC9003557; doi:10.3390/nu14071447)
Supplement: Supplementary file 1 [file nutrients-14-01447-s001.zip › Table S1.pdf]

Table S1. Food or beverage groups for analysis by trained panel and representative product of each group

Food or beverage group	Representative product
Baby foods	"My Vegetable Soup" (*Mi Sopita Verduras MINSAL®)*
Liquid dairy substitute drinks	Soy apple drink *(Manzana Ades®)*
Baby cereal	Wheat with sugar baby cereal *(Nestum Nestlé®)*
Sweet baby puree	Apple baby puree *(Gerber®)*
Savory baby puree	Meat with vegetable baby puree *(Naturnes Nestlé®)*
Sweet spread (carmel or *dulce de leche*)	*Manjar Nestlé®*
Non-nutritive sweetener in solution	Sucralose (Daily®)
Infant dairy formula	(Purita Fortificada MINSAL®)
Gelatin	Raspberry gelatin (Ambrosoli®)
Dehydrated soup	Asparagus soup (Maggi Nestlé®)
Dehydrated soup II	Vegetable soup (Años Dorados MINSAL®)
Powdered nutritional supplement (adult)	Vanilla Ensure (Abbott®)
Powdered nutritional supplement (infant)	Vanilla Pediasure (Abbott®)

MINSAL®: Products provided by the State through the Chilean Ministry of Health, in different Complementary Food Programs.
