# Supplementary material for: Sweetness of Chilean Infants’ Diets: Methodology and Description
Source: Nutrients. 2022 Mar 30;14(7):1447. doi: 10.3390/nu14071447 (PMC9003557; doi:10.3390/nu14071447)
Supplement: Supplementary file 1 [file nutrients-14-01447-s001.zip › Table S2.pdf]

Table S2. Reference products and solutions for sweet tastes

Solutions and reference products	Sweetness intensity value
Sucrose solution in distilled water concentration, 20 g / l	13
Sucrose solution in distilled water concentration, 50 g / l	33
Sucrose solution in distilled water concentration, 100 g / l	66
Whole milk (Soprole®)	12
Cookie (*galleta de vino McKay®)*	30
Marshmallow (Ambrosoli®)	60
Condensed milk (Nestle®)	88
