# Supplementary material for: Sweetness of Chilean Infants’ Diets: Methodology and Description
Source: Nutrients. 2022 Mar 30;14(7):1447. doi: 10.3390/nu14071447 (PMC9003557; doi:10.3390/nu14071447)
Supplement: Supplementary file 1 [file nutrients-14-01447-s001.zip › Table S3.pdf]

Table S3. Food groups

N°	**Food groups**
1.	Baby foods
2.	Sugars and candy
3.	Beverages
4.	Fast food chains
5.	Meat and substitutes (e.g., soy meat)
6.	Grains and bread
7.	Fruits
8.	Fats and Oils
9.	Vegetables / algae and mushrooms
10.	Eggs and egg products
11.	Dairy and substitutes (e.g., almond drink)
12.	Legumes
13.	Miscellaneous (e.g., nutritional supplement)
14.	Fish and shellfish
15.	Sauces, condiments and dehydrated soups
16.	Snacks
